# Effectiveness of Biodiversity Surrogates for Conservation Planning: Different Measures of Effectiveness Generate a Kaleidoscope of Variation

**DOI:** 10.1371/journal.pone.0011430

**Published:** 2010-07-14

**Authors:** Hedley S. Grantham, Robert L. Pressey, Jessie A. Wells, Andrew J. Beattie

**Affiliations:** 1 Key Centre for Biodiversity and Bioresources, Macquarie University, Sydney, New South Wales, Australia; 2 The Ecology Centre, University of Queensland, St Lucia, Queensland, Australia; Umea University, Sweden

## Abstract

Conservation planners represent many aspects of biodiversity by using surrogates with spatial distributions readily observed or quantified, but tests of their effectiveness have produced varied and conflicting results. We identified four factors likely to have a strong influence on the apparent effectiveness of surrogates: (1) the choice of surrogate; (2) differences among study regions, which might be large and unquantified (3) the test method, that is, how effectiveness is quantified, and (4) the test features that the surrogates are intended to represent. Analysis of an unusually rich dataset enabled us, for the first time, to disentangle these factors and to compare their individual and interacting influences. Using two data-rich regions, we estimated effectiveness using five alternative methods: two forms of incidental representation, two forms of species accumulation index and irreplaceability correlation, to assess the performance of ‘forest ecosystems’ and ‘environmental units’ as surrogates for six groups of threatened species—the test features—mammals, birds, reptiles, frogs, plants and all of these combined. Four methods tested the effectiveness of the surrogates by selecting areas for conservation of the surrogates then estimating how effective those areas were at representing test features. One method measured the spatial match between conservation priorities for surrogates and test features. For methods that selected conservation areas, we measured effectiveness using two analytical approaches: (1) when representation targets for the surrogates were achieved (incidental representation), or (2) progressively as areas were selected (species accumulation index). We estimated the spatial correlation of conservation priorities using an index known as summed irreplaceability. In general, the effectiveness of surrogates for our taxa (mostly threatened species) was low, although environmental units tended to be more effective than forest ecosystems. The surrogates were most effective for plants and mammals and least effective for frogs and reptiles. The five testing methods differed in their rankings of effectiveness of the two surrogates in relation to different groups of test features. There were differences between study areas in terms of the effectiveness of surrogates for different test feature groups. Overall, the effectiveness of the surrogates was sensitive to all four factors. This indicates the need for caution in generalizing surrogacy tests.

## Introduction

Most species have not yet been described and even for the minority that are known, data on spatial distributions are sparse and often unreliable. Further, knowledge of the processes that sustain biodiversity is rudimentary for most regions. To plan for representative protected areas therefore requires surrogates for biodiversity [1], [2]. When attempting to represent patterns of biodiversity in conservation areas, biodiversity surrogates used by planners include some of the better-known taxonomic groups, focal species, umbrella species, species assemblages, and various ecological classifications [2], [3], [4], [5], [6]. Methods directed to conserving biodiversity processes, though less common, are increasing [7], [8].

Surrogates can be roughly divided into taxonomic and environmental categories. Taxonomic surrogates are predominantly based on biological data, include the use of well-known groups of species such as birds, and are often extrapolated geographically using statistical techniques [9], [10]. Environmental surrogates are usually based on a mix of physical and biological data. They can be subdivided into two types: those based on discrete classes (often referred to as ecological classifications or land types); and surrogates where continuous data are analyzed directly in the selection of areas [see 11,12]. Ecological classifications have been widely used as surrogates in conservation planning [e.g. 13], [14], [15], [16], often with the assumption that they will represent large numbers of subsumed species [17]. They can reflect factors known to be important in determining the distributions of species and, compared with species data, can be mapped more consistently, quickly, and inexpensively across large areas [2]. They have been derived in many ways, the choices being guided by data availability, spatial scale, choice of data merging techniques, biogeography, and perceptions about the importance of particular variables in shaping biological distributions [e.g. 2], [13], [18], [19]. The economy and consistency of ecological classifications are weighed against several limitations, some long recognized by conservation planners [16]. These include patchy distributions of species within and between classes, especially for rare, locally endemic, and threatened taxa; the frequent absence of large compositional changes at mapped boundaries, and lack of information on important areas such as drought refugia and breeding sites that occur at finer spatial scales [16], [20], [21], [22], [23], [24], [25]. Many planners have compensated for the limitations of ecological classifications by using datasets composed of multiple surrogates [e.g. 2], [15].

Testing the effectiveness of ecological classifications as surrogates for other aspects of biodiversity can improve methods for developing new surrogates, and help planners to understand their unavoidable limitations. For biodiversity patterns, effectiveness refers to the ability of the surrogate to reflect the distribution of some other features of biodiversity. Methods for assessing effectiveness require measurement of surrogate performance relative to test features (i.e. other aspects of biodiversity that the surrogate is intended to represent), and can be loosely categorized as either pattern-based or selection-based. Pattern-based tests [e.g. 20], [26], [27] directly measure the spatial relationship between the surrogate and test features, but do not directly assess the outcomes of alternative conservation decisions. Selection-based techniques generally select notional conservation areas based on the surrogate, then measure representation, or likelihood of representation, of the test features in those areas [e.g. 24], [25], [28]. Selection-based methods therefore address conservation decisions [29] but have the relative disadvantage of assuming particular configurations of selected areas or probabilities of selection that are unlikely to match conservation action as it is realized on the ground. Systematic selections are rarely implemented entirely and without alteration [30]. Therefore, uncertainties in implementation could alter the apparent effectiveness of surrogates.

Different studies have reported widely varying results on the effectiveness of ecological classifications as surrogates [e.g. 4], [6], [20], [21], [23], [24], [25], [31], [32], [33], [34], [35], [36], [37], [38], [39], [40]. Variation in these results might reflect differences among studies in several key characteristics, such as, study area location, spatial extent, spatial resolution, type of surrogate, taxa (or other test features) used to evaluate surrogates, and analytical methods used to test surrogates. Each of these factors can be expected to influence results [1], [41]. Importantly, previous studies that have tested environmental surrogates have involved simultaneous variation in most or all of these factors, making it impossible to discern the influence of any single factor. It is therefore not surprising that a large body of work has produced variable results and few, if any, generalizations.

Our study used a rich data set as an opportunity to systematically assess the influence of four key factors, alone and in combination, in determining the apparent effectiveness of ecological classifications as surrogates. These factors were: (1) two study regions, (2) two surrogates, (3) five testing methods, and (4) six groups of threatened species as test features, against which we measured the effectiveness of the surrogates (Table 1 provides a full description of each of these factors). We used a subset of possible selection-based testing methods that have commonly been applied in the literature and vary in their assumptions, limitations and advantages.

**Table 1:** Four key factors analyzed to determine the apparent effectiveness of biodiversity surrogates.

Factor	Variables	Description
Study regions	1) Upper north east NSW	Located in north east NSW, Australia (Fig. 1)
	2) Lower north east NSW	Located in north east NSW, Australia (Fig. 1)
Surrogates	1) Environmental units	Classes were based on 4 environmental variables. There were 37 classes in upper north east NSW and 40 in lower north east NSW.
	2) Forest ecosystems	Classes were based on forest types and floristic/environmental variation. There were 96 classes in upper north east NSW and 95 in lower north east NSW.
Testing methods	Method 1- Incidental representation (measuring median target achievement)	Areas were first selected to achieve representation targets for the surrogate, then effectiveness was measured as the median representation target achieved incidentally for the test feature group.
	Method 2- Incidental representation (measuring percentage of features to target)	Similar to method 1 except effectiveness was measured as the percentage of test features with targets fully achieved.
	Method 3- Species accumulation index measuring median target achievement	Areas were selected progressively to achieve representation targets for the surrogate, then effectiveness was measured based on the increase in the median achievement of test feature targets in relation to median target achievement by random selection of areas and by “optimal” selection using the test features themselves instead of the surrogates.
	Method 4- Species accumulation index measuring percentage of features to target	Similar to method 3 except effectiveness was measured as the percentage of test feature with targets fully achieved.
	Method 5- Correlation of irreplaceability	Irreplaceability is an index of the conservation value of areas in contributing to conservation targets. Irreplaceability patterns of areas based on targets for surrogates were correlated with those based on targets for each test feature group.
Test feature groups	1) All test features	412 species/sub-species in upper north east NSW and 298 in lower north east NSW.
	2) Mammals	77 species/sub-species in upper north east NSW and 82 in lower north east NSW.
	3) Birds	42 species/sub-species in upper north east NSW and 31 in lower north east NSW.
	4) Reptiles	91 species/sub-species in upper north east NSW and 175 in lower north east NSW.
	5) Frogs	43 species/sub-species in upper north east NSW and 31 in lower north east NSW.
	6) Plants	159 species/sub-species in upper north east NSW and 79 in lower north east NSW.

The combination of these four factors generated 120 assessments of surrogate effectiveness.

## Results

**Figure 1:**
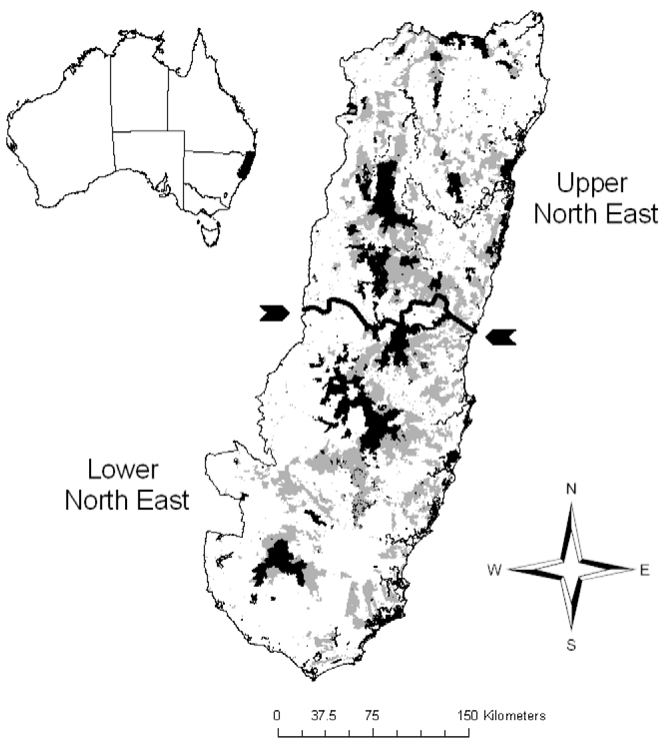
Study areas showing existing reserves and public forests in north-eastern New South Wales. Existing reserves are shown in black. Public forests open for negotiation and further conservation management are in grey. The configuration is from 1998, prior to the Regional Forest Agreement that extended the reserve system. The region was divided into two study areas–upper and lower–along the dark line, also indicated by arrows.

**Figure 2:**
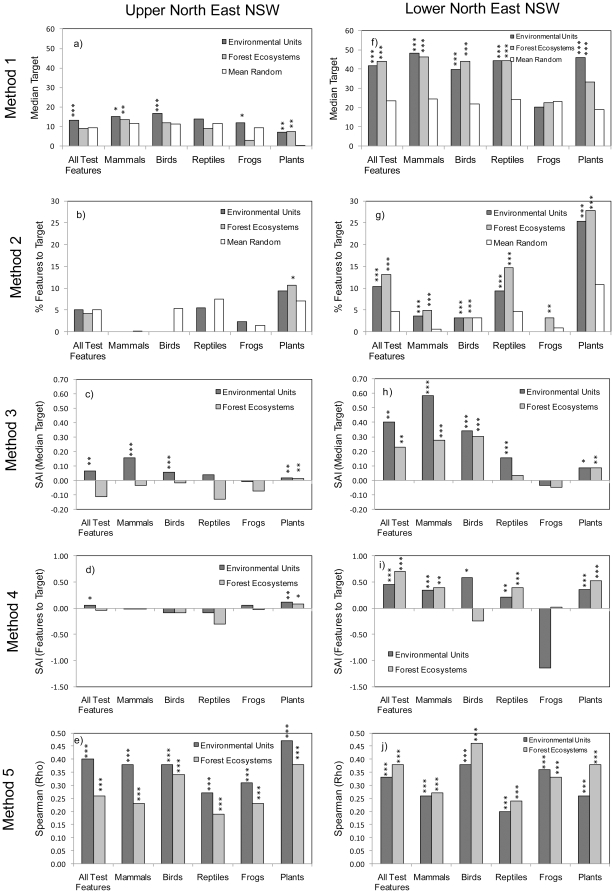
Summary of all results, showing effectiveness estimates (absolute values) arranged by study area and testing method. Note that absolute values are not comparable between testing methods. Asterisks indicate significance levels (*** p<0.001; ** p<0.01; and * p<0.05) for comparisons of the surrogate's effectiveness versus a null-distribution of randomly selected areas (for methods 1–4) randomly paired planning units (for method 5). Method 1-incidental representation measuring median target achievement; method 2- incidental representation measuring percentage of features with targets fully met; method 3- species accumulation index (SAI) measuring median target achievement; method 4- species accumulation index (SAI) measuring percentage of features with targets fully met; and method 5- correlation of summed irreplaceability values.

In each of our two study areas (Fig. 1), we applied 60 tests of effectiveness (see Table 1), involving 2 surrogates, 6 test feature groups (including all groups combined), and 5 testing methods. Overall results for the upper north-east of New South Wales are in Fig. 2a–e and those for the lower north-east are in Fig. 2f–j.

### Overall performance of surrogates

Environmental units were more effective than forest ecosystems in 33 instances compared with forest ecosystems that were more effective in 22 instances, and 5 had similar results (Fig. 2.). Differences in values, however, were often relatively small. The surrogates were more effective than random selections of areas (methods 1–4, p<0.05) or showed significant correlations of summed irreplaceability values (p<0.05) in 79 out of 120 cases (Fig. 2).

Test features were generally poorly represented by, or correlated with, surrogates (Fig. 2.). Across surrogates and test feature groups, the highest values from Method 1 (median percentage target achieved) were 17 in the upper north-east and 48 in the lower north-east (maximum possible values 100). The highest values for Method 2 (percentage of features with targets achieved) were 11 and 28 (maximum possible values 100). For Method 3 (species accumulation index based on median target achievement) the values were 0.16 and 0.58, and for Method 4 (species accumulation index based on percentages of targets achieved) the values were 0.12 and 0.70 (maximum possible values 1.0). For Method 5 (correlations of summed irreplaceability), the highest values were 0.47 in the upper north-east and 0.46 in the lower north-east (maximum possible values 1.0). Overall, values of effectiveness were much lower than maximum.

**Figure 3:**
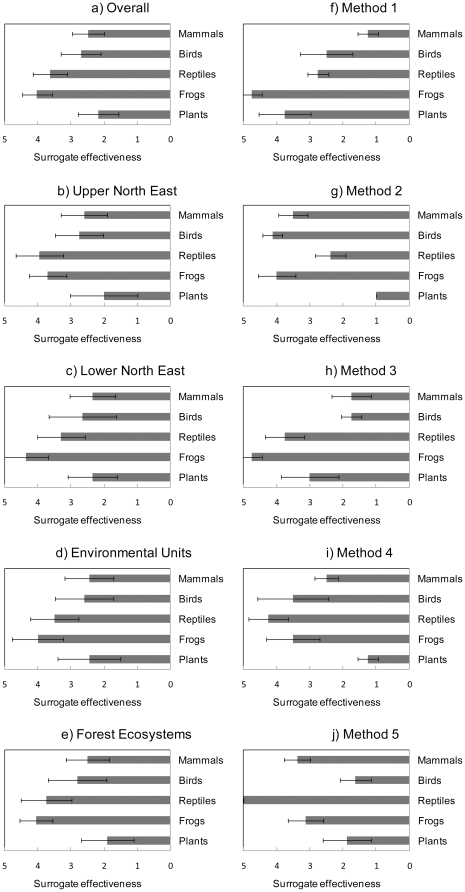
Mean rankings of test feature groups (with 95% confidence intervals). A rank of 1 indicates highest surrogate effectiveness and 5 indicates lowest. Results are grouped across (a) all tests, (b and c) two study areas, (d and e) both surrogates and (f–j) each method. Method 1- incidental representation measuring median target achievement; method 2- incidental representation measuring percentage of features with targets fully met; method 3- species accumulation index measuring median target achievement; method 4- species accumulation index measuring percentage of features with targets fully met; and method 5- correlation of summed irreplaceability values.

Overall ranking of the test feature groups showed that surrogates were most effective for plants (Fig. 3a). This was also the case for comparisons considering the upper north-east region separately (Fig. 3b) whereas, in the lower north-east, mammals and plants were equally best represented by surrogates (Fig. 3c). Effectiveness of both environmental units and forest ecosystems was higher for plants and mammals than for other test feature groups (Fig. 3d–e). In all five comparisons that combined testing methods (Fig. 3a–e), the surrogates were least effective for frogs and reptiles.

### Comparison of study areas

The two study areas showed differences in surrogate effectiveness values overall and for environmental units and forest ecosystems (Fig. 2). Values were generally higher in the lower north east. For the same surrogate, testing method and test feature, the values were higher in the lower north east in 51 out of 60 cases and 2 cases were equal. In the upper north east, environmental units were more effective than forest ecosystems in 24 out of 30 cases and 2 cases were equal (Fig. 2a–e). In the lower north-east, forest ecosystems were more effective than environmental units in 20 out of 30 cases and 1 case was equal (Fig. 2f–j). The two regions produced similar ranks across the test features in a majority of the test settings. There were no examples of ranks at opposite extremes (i.e. a rank of 1 in one study region and a rank of 5 in the other) for the same test. We found moderately diffuse correlations between these ranks, yielding Spearman's correlation *r_s_* = 0.58 (p<0.001) and Kendall's concordance coefficient W = 0.785 (p<0.001).

### Comparison of methods

**Figure 4:**
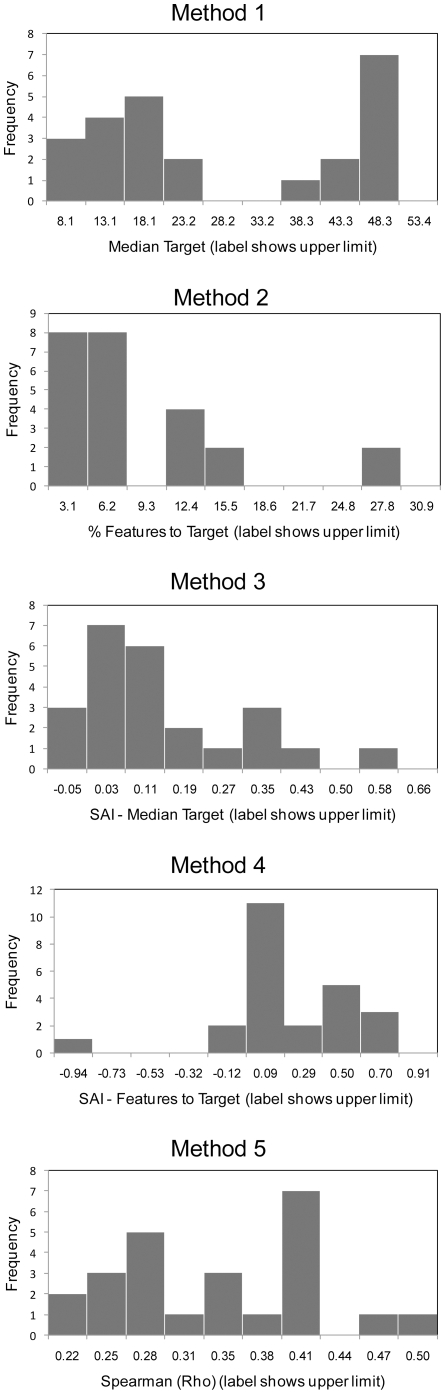
Histograms of 24 absolute values for each testing method. Method 1- incidental representation measuring median target achievement; method 2- incidental representation measuring percentage of features with targets fully met; method 3- species accumulation index measuring median target achievement; method 4- species accumulation index measuring percentage of features with targets fully met; and method 5- correlation of summed irreplaceability values.

We applied each testing method to 24 combinations of study area, surrogate type, and test feature group. Different testing methods produced different rankings of test feature groups (Fig. 3f–j).The methods also produced varying distributions of results (Fig. 4). With the 24 results for each testing method ranked, Spearman correlation coefficients for the ranks (*r_s_*) of one method against another were mixed. There were five significant correlations ranging from 0.44 to 0.71, with the strongest between methods two and four, and five non-significant correlations (Table 2).

**Table 2:** Relationships between effectiveness estimates from five alternative testing methods.

	Method 2	Method 3	Method 4	Method 5
Method 1	0.44*	0.72***	0.50*	−0.1
Method 2		0.46*	0.71***	0.14
Method 3			0.61**	0.36
Method 4				0.07

Effectiveness values were ranked on their native scale for each method (for each method n = 24 based on different combinations of study area, surrogate, and test feature group) and compared using Spearman's rank correlation coefficient. Asterisks indicate significance levels (*** p<0.001; ** p<0.01; and * p<0.05).

## Discussion

This is the first time the individual effects of four key factors; 1) choice of surrogate, 2) test features 3) study area, and 4) testing method have been considered when evaluating surrogates. We found that the effectiveness of surrogates was sensitive to all of them. This raises important issues to be addressed if the concepts of surrogacy and effectiveness are to contribute meaningfully to data collection and conservation decisions.

### Influence of surrogate type

We found that environmental units were more effective surrogates than forest ecosystems in the upper north east but this result was reversed in the lower north east. Differences in surrogate values, however, were relatively small. This contrasts with the findings from the same region by Ferrier et al. [6], [42], [43], [44] who concluded that forest ecosystems were far more effective than environmental units. These differences underline the sensitivity of apparent surrogate effectiveness to the study design. For example, Ferrier et al. tested surrogates against a far wider variety of species including threatened and non-threatened invertebrates, vertebrates and plants which necessarily included groups of organisms that differed greatly in mobility and habitat preferences [45]. They used the species accumulation index (implemented as our method 4) as one of their testing methods, but did not consider the practical constraints of existing reserves (e.g. over representing particular species and habitats). Also, their selections were based on survey sites rather than planning units, and they counted species as represented if they occurred in a single selected survey site, rather than addressing species-specific targets.

### Influence of test features

Threatened species were important as test features both because of the need for protection and their generally restricted distributions, making them more likely than other species to be missed by conservation areas selected only with ecological classifications as surrogates. Our study demonstrated the both surrogates performed well for plants. This is not surprising given floristic components were part of its classification. The environmental units might have performed well for plants due to soil fertility, a variable used to develop the classification, is a factor that can also influence plants distributions [46]. Neither of our surrogates performed strongly for our test features. Values for all methods were generally well below their potential maxima. While not surprising, this highlights a dual problem for conservation planners: distribution data are often relatively poor for threatened species [47] and ecological classifications can be relatively ineffective as surrogates for threatened species [24], [48]. This result was also partly due to a limited number of areas being required to achieve targets for the surrogates. When selecting areas to achieve targets for the test features, we found that large areas are needed for some taxa.

Similar to our study, Araújo et al [49] found threatened frogs and reptiles most likely to be missed by conservation planning based on surrogates, although they tested different kinds of surrogates. For the ecological classifications tested here, there are at least two possible reasons why frogs and reptiles would be missed more often than other taxa. First, on both our study regions, the predicted distributions of frogs and reptiles were much smaller on average than those of mammals and birds. The differences might be real or could reflect the influence of fewer field records and more limited observations of habitat associations of frogs and reptiles in the region [50]. Overall, rarer features are more likely to be missed by areas selected to represent targets for surrogates [24], [25] despite, in our study, correspondingly smaller targets that were easier to achieve. A second possible reason for surrogates being relatively ineffective for frogs and reptiles is that these organisms are distributed in response to habitat characteristics that are poorly reflected by environmental units or forest ecosystems. Frog species, for example, are often confined to specialised micro-habitats and have complex life histories encompassing both aquatic and terrestrial phases [51]. Similarly, invertebrates have been found to be generally poorly represented by various biodiversity surrogates due to their often specific habitat characteristics [e.g. 21], which is significant given that they comprise the majority of biodiversity.

### Influence of study area

Regions with relatively comprehensive datasets have been proposed as test beds of surrogate effectiveness, providing lessons for regions with poorer data [42], [52]. This approach is practical and intuitively appealing, but might be limited if the results from one region are difficult to generalise. Our results showed some differences between study areas. First, values produced by the testing methods were generally higher in the lower north east. Second, we found that the relative effectiveness of our two surrogates differed between study areas. The two regions however, showed only slight differences in the rankings of effectiveness across test groups for each method and the two regions ranking were significantly correlated. There are two likely reasons for any study region effects. First, there were approximately 25% more test features in the upper north-east (including almost twice as many plant species) and they were generally more narrowly distributed, making them more likely to be missed, despite correspondingly smaller targets that were easier to achieve. A second reason is that initial target achievement for environmental units varied markedly between study areas. For methods 1–4, the number of areas selected to achieve targets for environmental units was the benchmark for comparing the two surrogates. Corresponding to differences in initial target achievement, only 11% of the area available for conservation was required to achieve targets for environmental units in the upper north-east, but 30% in the lower north-east. This large difference directly affected values of incidental representation of test features, measured at the end of selections, with values generally lower in the upper north east.

Any differences between these regions are noteworthy given the proximity of the study areas, and their close similarity in terms of patterns of tenure and land use, physical environment, biota, and methods and scales for mapping surrogates, surveying the biota, and predicting species distributions. These differences also highlight potential inaccuracies of predicting species and habitat distributions outside of their dataset range.

### Methods for quantifying ‘effectiveness’

Previous studies have shown that the choice of testing method can influence the apparent effectiveness of surrogates [e.g. 28], [39]. Our methods produced different distributions of values, and hence convey more or less optimistic pictures of surrogate effectiveness. More importantly, we found that some methods had different rank orders of results in relation to their effectiveness for different test. Previous work points to further factors that might interact with testing method to influence the apparent effectiveness of surrogates. These include the extent of the study region and size of planning units [41] and the size of surrogate targets which change patterns of irreplaceability and selections of areas [e.g. 53].

Several studies have argued that measuring the performance of surrogates requires the selection of notional conservation areas based on a surrogate, followed by measuring species representation compared with that obtained from selections generated at random [29], [54], [55]. For example, Rodrigues et al. [55] state that “The relevant question in a surrogacy test is, therefore, what is the extent to which areas selected for surrogates capture the target features?” We are less confident that conservation science has converged on a single, effective method. There are three main reasons. First, the respective assumptions, strengths and limitations of selection-based and pattern-based testing methods remain poorly understood. Second, different plausible methods produce different and sometimes conflicting results. For example, even within the species accumulation index, the extent of ‘representation’ relative to targets can be quantified in alternative ways (median % of targets reached and percentage of features with targets reached in this study; or, as employed in its simplest form, as a binary target of represented or not as employed by Rodrigues et al. [55]). Rankings of values from the two forms of species accumulation index used here were imperfectly correlated. Third, the data-dependence of results from different methods has been poorly explored by applications to multiple regions and planning situations.

In our study, incidental representation (methods 1 & 2) demonstrated how a notional conservation system based on surrogates might contribute to the protection of biodiversity such as threatened species, considering the existing conservation system and its associated environmental bias. A limitation of this approach was its inability to measure effectiveness progressively as more areas were selected. The species accumulation index (methods 3 & 4) overcomes this limitation by integrating the relative performance of surrogates and “optimal” selections as areas are progressively added to the conservation system. Both methods, however, involve two important assumptions. The first is that the selected areas are indicative of the composition and configuration of future conservation areas on the ground. This is very unlikely given the socio-economic and political forces that shape actual conservation systems in our study areas [56] and elsewhere, even when systematic methods underpin planning. A second assumption is that single sets of selected areas are adequate indicators of incidental representation or the species accumulation index. In most regions, there are many possible ways of assembling areas into representative systems [57], [58]. It is therefore important to know how the results of selection-based methods might change between alternative sets of areas.

We attempted to overcome this second limitation by using correlations between patterns of summed irreplaceability (method 5) based on the surrogates and test features, effectively considered all possible ways of assembling systems of conservation areas [25], [28], [59]. This method assumes that irreplaceability indicates the likelihood of areas being selected for conservation, or that choices between optional areas with similar irreplaceability values will be resolved randomly. Given real-world constraints and preferences this is unlikely and the actual resolution of options will probably be region-specific and determined to some extent by socio-economic factors. Also, it may be helpful to consider features of the two distributions of irreplaceabilities beyond only their single linear correlation coefficient, for example, if conservation actions can only cover a small proportion of sites, then we may be more concerned with the performance of the surrogate in identifying or ranking the sites with highest irreplaceability for the test feature, which could coincide with either low or high correlation across the vast majority of locations.

A further critical aspect of selection-based methods is significance testing of the results. Like other authors, we used random selection of areas as a null model to compare the outcomes of incidental representation and the species accumulation index. Random selections are useful as a baseline because they are likely to sample the physical and biological variation within a region and provide a neutral baseline for comparison with representation of biodiversity from deliberate selections [see 55 for discussion]. However, an alternative null model might involve simulating conservation involving realistic forms of bias, for example, selecting areas least valuable for extractive uses to approximate widely observed residual conservation systems [60]. Another informative baseline might be a conservation system designed by expert-opinion rather than data sets in conservation planning software [61]. There appear to be no studies of the relative performance of selection-based methods against these alternative null models.

### A future for environmental surrogates?

Our results demonstrate that ecological classifications have some, albeit limited, value as surrogates for threatened species, as others have found in earlier studies [e.g. 24], [48]. Ecological classifications are often used in conservation planning as generalized, coarse filter surrogates. Their perceived role is to compensate for the spatial and taxonomic biases inherent in any species-based data sets [62], the lack of congruence between many taxa [e.g. 63], [64], the likelihood of missing higher-level interactions between species and their environments [33], the large cost of obtaining new species data [65], and other limitations [66]. However, some authors have strongly advocated the use of taxonomic surrogates instead of environmental surrogates, even in light of their expense and limitations, if alternatives (environmental surrogates) are too coarse or lacking in biological justification [67]. Surprisingly, we are only aware of a few studies that have compared taxonomic and environmental surrogates. Carmel & Stroller-Cavari [31] found the two types to be similarly effective. Rodrigues & Brooks [55] applied a meta-analysis of 27 studies and found stronger support for taxonomic surrogates. Nonetheless, their selection of testing methods was limited to species accumulation indices, which they considered to be most robust *a priori*. Our study suggests the relative merits of different testing methods are unresolved. Further, most of the tests of environmental surrogates in their meta-analysis came from one region (north-eastern New South Wales) following the work of Ferrier and Watson [44]. Our results from the same area have demonstrated the potential for these results to be region-specific. We therefore consider the choice between environmental and taxonomic surrogates to be an open question. Perhaps this debate also over-emphasises the distinction between taxonomic and environmental surrogates, rather than acknowledging the extreme heterogeneity of surrogacy value offered by choices within these two broad classes, and the need for any choice to be based on ecological and biogeographic understanding of the relationship between a surrogate and the underlying conservation objectives. New methods are emerging that make the best use of all available data in a region when developing surrogates for biodiversity, such as, generalised dissimilarity modelling [12].

### How to understand surrogate effectiveness?

How can we learn from surrogacy tests? Meta-analysis across surrogate tests might yield generalizations and identify the main factors underlying variability in results, thereby refining predictions about surrogates and methods for testing them. To identify the influence of any one factor on the effectiveness of environmental surrogates, meta-analysis will have to draw on sufficient studies to adequately represent variation in other factors. The difficulty here is highlighted by the number of possible combinations of study area, extent, resolution, surrogate type, test features, and analytical method. For example, we are aware of perhaps 20 different testing methods currently applied to environmental surrogates. Complementary to meta-analysis, we identified the individual influence of four factors; study area, surrogate type, test features, and testing method, likely to influence surrogate effectiveness by systematic explorations of their variations within a well-studied system. The importance of this case study is in its rigorous demonstration that all these factors influence the measured effectiveness of surrogates. Yet none of these factors was taken into consideration in the only meta-analysis applied to environmental surrogates to date [55]. Rodrigues & Brooks [55] standardized their comparisons by using a single version of the species accumulation index, and therefore restricted their analysis to 27 studies out of several hundred. Any future meta-analysis should attempt to broaden the number of factors considered. Furthermore, we believe there is a clear need for more research to better understand the alternative methods for quantifying effectiveness, in terms of their advantages, limitations and assumptions. We also recommend that insights into surrogates could be gained from reviewing aspects of the ecology and biogeography of species that both support the use of surrogates and explain their inevitable limitations.

Conservation planning is a dynamic process and planners must continually make decisions about the allocation of scarce resources. In relation to biodiversity data, planners are faced with questions including: what decisions to make using existing data; which data to ignore; and what might be the most cost-effective types of additional data. Planners also have to choose between making decisions with available data or waiting for better data while risking the loss of important areas [68]. Further choices concern the marginal benefits of data collection in different regions. Conservation science has provided few solutions to these practical problems [69]. Addressing this gap requires studies that place surrogacy measures firmly in the context of decision-making processes and resources available. We need new novel methods that explicitly trade-off the value of new data and knowledge against the implementation of more decisive conservation action.

## Materials and Methods

### Study areas

Our study areas were in north-eastern New South Wales, Australia (Fig. 1): the upper and lower north-east. These were the boundaries used for a conservation planning process in 1998, called the Regional Forestry Agreements, that established extensive new conservation reserves [2]. We used the configuration of tenures as they existed in 1998 (before the establishment of new reserves), because this enabled us to consider a large number of potential areas for conservation management, and to assess the effectiveness of surrogates against actual conservation targets used in the forestry reform process. At that time, nearly 20% of the study area was covered by some sort of conservation management with around 10% in strict reserves. The two study regions are very similar in their patterns of tenure, land use, physical environment and biota. Any differences that we find in apparent effectiveness of surrogates in these highly similar regions will therefore have large implications for our ability to generalise from one region to another, considering that differences between most study regions are far greater.

### Biodiversity surrogates

Our two biodiversity surrogates, forest ecosystems and environmental units, have both been used extensively for conservation assessments in the study areas but differ strongly in their derivation and resulting spatial distributions. Consequently, they are not merely subdivisions of one another and their boundaries rarely coincide [5], [70]. Eighty-one environmental units classes were previously derived by combining four environmental variables: mean annual rainfall, mean annual temperature, soil fertility (based on geology) and slope [60]. These were mapped across all tenures and land uses, so pre-deforestation extents were known, and then intersected with remaining vegetation. They were also derived across a larger extent than our study regions, so only 37 classes were analyzed in the upper north east and 40 in the lower north east. Some 157 forest ecosystems classes were originally derived by subdividing or amalgamating forest types [71] according to variation in floristic composition and environmental variables. Their occurrence was predicted across gaps in mapping of forest types, including deforested land, in relation to environmental variables [6]. After trimming the extent of forest types to our study regions we analyzed 96 classes in the upper north east and 95 in the lower north east.

### Species data and test features

To test the effectiveness of the surrogates, we used data on forest-dependent plant and animal species listed as threatened under the New South Wales Threatened Species Conservation Act (1995) or the Commonwealth Endangered Species Protection Act (1992) or nominated by experts as requiring conservation action [72]. Data for a given taxon consisted either of locality records alone (point data), or predicted distributions from previous modeling in relation to physical variables and vegetation structure [5]. Distribution models yielded probabilities of occurrence, and the previous work converted these to predicted densities in two steps, both with involvement of experts on each taxon [72]. First, the ranges of predicted probabilities were subdivided into four habitat qualities: core, intermediate, marginal, and unsuitable. Second, an estimated density, such as number of breeding females per km^2^, was assigned to each quality class for each species. Most animal species and some plant species were divided into geographically distinct populations, on the basis of putative dispersal barriers likely to prevent re-colonization of vacant habitat. These populations were treated as separate ‘species’ in subsequent analyses. We removed from consideration any species for which conservation targets were already achieved, or that occurred only in planning units excluded from our analyses (see below), leaving 159 plant and 235 animal species in the upper north-east (total 394), and 79 plant and 219 animal species in the lower north-east (total 298). Animals had much larger distributions than plants. To enable comparisons among alternative sets of test features, we grouped species into six test feature groups: all species combined, mammals, birds, reptiles, frogs, and plants.

Our test features provide insights into the effectiveness of ecological classifications as surrogates for species of conservation interest, based on data quality that is rarely available for threatened species. Tests of ecological classifications as surrogates for target taxa that are threatened or of conservation concern might yield lower estimates of effectiveness than for more widely distributed test features, as expected intuitively and seen in empirical studies [e.g. 24], [25]. However, these are the species that, if missed by conservation areas focused on surrogates, will have the poorest prognoses for persistence, so are justifiably a focus of surrogate testing. In any case, the general relevance of this study is underlined by our test features varying widely in distribution from highly restricted to widespread species within each taxonomic group. Also, each group contained species only with locality records (systematically under-estimating true presence) and others with distribution models that balanced errors of omission and commission [62].

### Conservation targets

Our three analytical approaches for testing surrogates, below, were based on reserve selection procedures and required conservation targets for both surrogates and test features. For the surrogate features, we set targets at 15% of the estimated pre-deforestation extent of each forest ecosystem and environmental unit, consistent with national policy for forestry reform when the data were compiled [73]. Targets for each threatened animal and plant species had previously been established [72], [74]. Targets for animals were calculated with a formula that related life-history parameters to area requirements for viable populations [72]. For plants with distribution models, area targets were based on demographic traits, likelihood of stochastic events, and expert opinion [74]. Plant species without distribution models were given targets for numbers of locality records according to listed threat status and assessments of conservation priority [72].

### Established reserves and planning units

Given the focus of the forest reform process on unreserved public lands, our analyses excluded both private land and existing reserves. The unreserved public lands had previously been subdivided into polygons forming planning units-the building blocks of potential conservation areas that are assessed and compared by decision support software [30]. Most of the planning units were defined as forestry management compartments, averaging 200 ha [2]. For each planning unit, we recorded the extent or number of locality records of each forest ecosystem, environmental unit, and species. We excluded planning units that were not fully covered by all surrogate data (environmental units were less extensively mapped than forest ecosystems). The planning units defined as ‘available for conservation management’ numbered 6,712 in the upper north-east and 7,021 in the lower north-east. If our exclusions of planning units made some targets unachievable, we reduced targets to match the extent or number of records in our data set. All analyses below were based on the portions of targets not already achieved in established reserves. Therefore the analyses consider the relative effectiveness of surrogates in achieving the remainder of species targets, given the fixed contribution from existing reserves.

### Testing methods

All five methods (Table 1) were based on selection, or likelihood of selection, of planning units as notional conservation areas to complement established reserves. We used the C-Plan software system [75] to: (1) select sets of planning units that met the targets for surrogates and test features; and (2) estimate the summed irreplaceability value of each planning unit [57]. The five methods represent three alternative analytical approaches to measuring the effectiveness of surrogates: incidental representation, species accumulation index, and correlation of summed irreplaceability values. For the first two of these approaches, the level of representation was quantified in two ways: (1) median % achievement of targets from the distribution of target achievement values of all taxa in the test set: and (2) % full achievement of targets, giving the percentage of taxa in the test set for which targets were fully achieved.

When selecting reserves based on the surrogates within each study area, we first performed iterative selection of planning units, based on summed irreplaceability [57], until all targets were met for environmental units. Achieving the targets for forest ecosystems required more planning units. However, for comparability of effectiveness at the same level of conservation ‘effort’, we terminated selections for forest ecosystems in each study area at the number of planning units required to achieve targets for environmental units (687 in the upper north east, 1666 in the lower north east).

The first analytical approach is termed ‘incidental representation’ [28]. We selected sets of planning units to achieve targets for each surrogate, and then measured how well species targets had been achieved incidentally in these planning units. For each test feature group, we used two alternative measures of incidental representation. For method 1 we used the median percentage target achievement for test features. For method 2 we used the percentage of test features with targets fully met. Higher values indicated greater effectiveness of the surrogates. We tested the significance of the results by randomly selecting 1000 times the same number of planning units needed to achieve surrogate targets and then measuring incidental representation based on each of the 1000 random sets (median percentage target for method 1 and percentage of features with targets fully met for method 2). We then compared the observed surrogate value to the distribution of values from random selections to determine significance [see 76]. The comparisons to random distributions of values were performed as post-hoc tests, and did not enter into the calculation of the reported value for effectiveness of the surrogates. Also, the calculations did not involve comparison to the best-possible values of surrogacy given *N* planning units selected to meet the targets for the surrogate (but see below).

The second analytical approach was the species accumulation index [6], [44]. We selected planning units iteratively, again using summed irreplaceability, to achieve targets for each surrogate, terminating selections for both surrogates at the number of areas needed for environmental units. This produced surrogate accumulation curves, relating targets of features incidentally achieved within each test feature group (vertical axis) to the number of planning units selected (horizontal axis). For method 3 we measured median target achievement within the test feature group. For method 4 we used the percentage of features in the test feature group with targets fully met. We then produced “optimal” accumulation curves by iteratively selecting planning units to achieve targets for the test features directly, ignoring surrogates. These curves represent hypothetical, best-possible results for the surrogate curves.

The species accumulation index reflects the closeness of the surrogate derived curve to the optimal curve and its distance from a random curve. It is calculated as (*s*–*r*)/(*o*–*r*), where *s* is the area under the surrogate curve, *o* the area under the optimal curve, and *r* the area under a mean random curve. We derived 1000 random curves by iteratively selecting areas at random up to the number required to achieve targets for environmental units, and repeating this 1000 times. For each of these sets of random selections, we measured the median target achievement and percentage of targets achieved for each test group and used the mean of these values across the 1000 randomisations to calculate the respective version of the index. Higher values indicate more effective surrogates [42]. Negative values occur when the surrogate curve is generally lower than the random curve, i.e. when planning units selected based on the surrogate achieve smaller gains for test feature targets than do randomly selected planning units. For each surrogate and test feature group, we calculated the significance of the area under the surrogate curve as the proportion of the 1000 random curves that had larger areas under them. This approach therefore directly incorporates comparisons to both: (1) optimal or best-possible results based on the test features themselves; and (2) null surrogacy values based on random selections.

The third analytical approach (implemented in Method 5) used the correlation of summed irreplaceability values. For each surrogate and test feature group, we estimated the summed irreplaceability [57] of each planning unit. This is a measure of the importance of each planning unit to achievement of targets for a feature group. Specifically, it estimates the sum, across all features, of the planning unit's irreplaceability with respect to achieving the target for each of the features separately. Minimum values were zero. The maximum value was equal to the number of features found in each planning unit. We calculated the Spearman coefficient of rank correlation between summed irreplaceabilities of all planning units for surrogates and groups of test features. Stronger correlations indicated more effective surrogates or, specifically, more spatial overlap between areas important for achieving surrogate targets and those important for achieving targets for test features. We tested for significance by pairing X and Y variables at random 10,000 times. Analytically, this involved randomizing the order of observations in the second column for each comparison. The null hypothesis was that the observed coefficient was zero [76]. For all comparisons, we used comparable combination sizes [57] for surrogates and test features.

### Ranking of test feature groups

We ranked mammals, birds, reptiles, frogs, and plants relative to each other (giving rankings from 1 to 5), according to surrogate effectiveness in the 2 study areas, for the 2 surrogates and for each of 5 testing methods. A rank of 1 indicated the group for which the surrogate was most effective. For comparison of test features, this gave us overall 20 sets of rankings, 10 sets for each study area, 10 sets for each surrogate, and 4 sets for each method. For a comparison of study regions it gave us 50 sets of rankings. Across all rankings and for subsets of rankings we compared test feature groups according to their mean ranks and 95% confidence intervals. For the study region comparison the 50 sets of rankings were paired according to each method, each surrogate and each test feature group. We used the Spearman's rank correlation coefficient to compare the relative ranks and tested the significance of correlations with randomization, as for Method 5, above. We also compared the ranks using Kendalls concordance coefficient.

### Comparison of methods

Comparisons of testing methods involved 24 sets of rankings (each method applied to 2 study areas, 2 surrogates and 6 test feature groups, including all groups combined). We used the Spearman's rank correlation coefficient to compare the relative ranks across methods and tested the significance of correlations with randomization, as for Method 5, above.
